# Supplementary material for: Spatial Uncertainty in Modeling Inhalation Exposure to Volatile Organic Compounds in Response to the Application of Consumer Spray Products
Source: Int J Environ Res Public Health. 2021 May 17;18(10):5334. doi: 10.3390/ijerph18105334 (PMC8157054; doi:10.3390/ijerph18105334)
Supplement: Supplementary file 1 [file ijerph-18-05334-s001.zip › ijerph-1207552-supplementary.pdf]

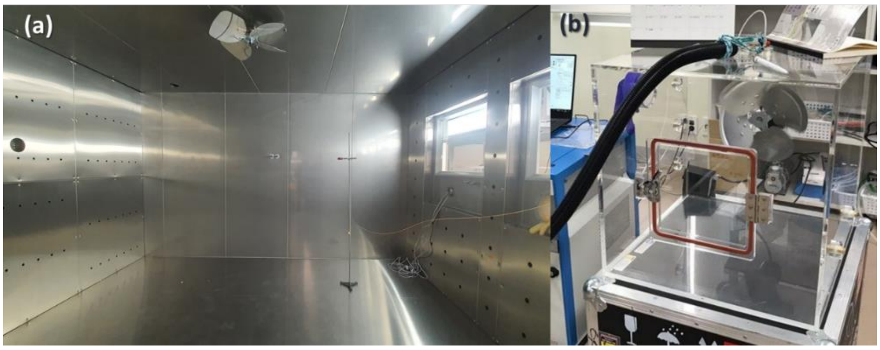

Figure S1 (a) Interior of the room-size chamber and (b) the acrylic chamber used in this study.

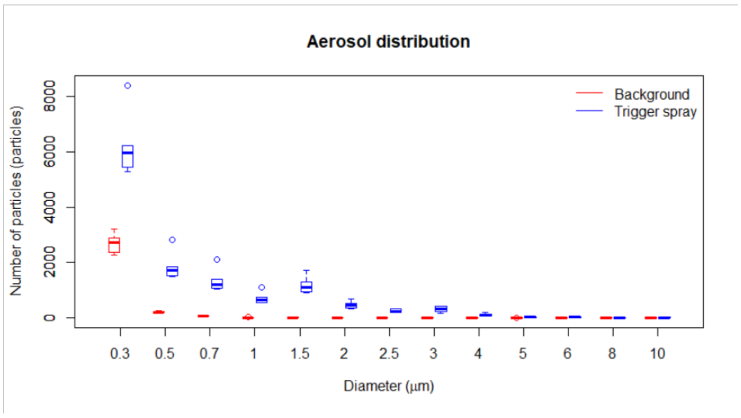

Figure S2 Size distribution of aerosols from an application of a trigger spray and the background of the room-sized chamber.

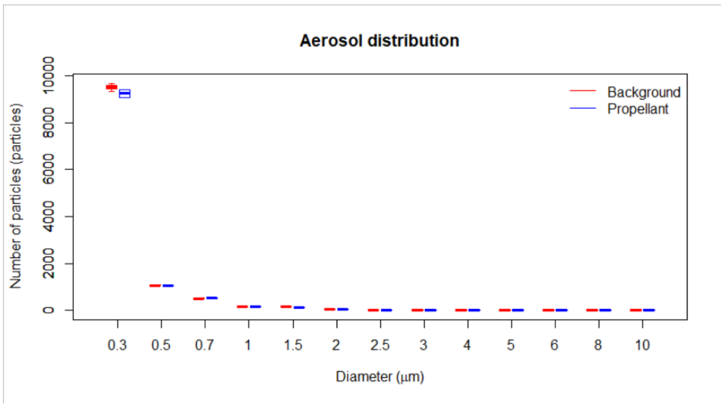

Figure S3 Size distribution of aerosols from an application of a propellant spray and the background of the room-sized chamber.
